# An Aberrant Case of Foreign Body Granuloma in the Left Forearm Due to an Impacted Piece of Glass Bangle

**DOI:** 10.7759/cureus.40925

**Published:** 2023-06-25

**Authors:** Rohit R Joat, Avinash Dhok, Kajal Mitra, Suresh Phatak, Gajanan K Wattamwar

**Affiliations:** 1 Radiology, NKP Salve Institute of Medical Sciences, Lata Mangeshkar Hospital, Nagpur, IND

**Keywords:** post traumatic granuloma, ultrasonography of granuloma, radiography of granuloma, foreign body granuloma, foreign body

## Abstract

Foreign body granuloma is an inflammatory tissue reaction around retained foreign bodies after penetrating trauma. The granulomatous reaction is a natural mechanism of the body to heal wounds by restricting the damage done by a pathological agent and containing the pathological agent to the wound site only. Here we present a classic case of a foreign body granuloma on the dorsal aspect of the forearm. This case was evaluated radiologically on high-frequency ultrasound and X-ray imagining and was operated on afterward. A piece of broken glass bangle was found inside the granuloma.

## Introduction

In granulomatous reactions, there is either resorption of organic material or sequestration of inorganic material [1]. If complete absorption of the foreign body takes place, it will lead to the stoppage of the granulomatous reaction. But if this does not happen, then a capsule is formed around the foreign body, leading to the cessation of immune reaction. Finding foreign bodies is always a challenge for surgeons because most of them are initially missed. In the case of wooden foreign bodies, only approximately 15% are seen on radiographs, leading to a missed diagnosis or wrong diagnosis. Trauma or iatrogenic injuries are also important causes of foreign bodies. Various complications are known to be associated with them, some of them being hemorrhage, septicemia, and abscess [2].

## Case presentation

A 50-year-old female presented with complaints of swelling over the left distal forearm along with a scar over the wrist region. She had a history of a road traffic accident two years prior with an injury to her left hand. She did not seek treatment for her lacerated wound and recalled a history of having her bangle broken during the accident.

**Figure 1:**
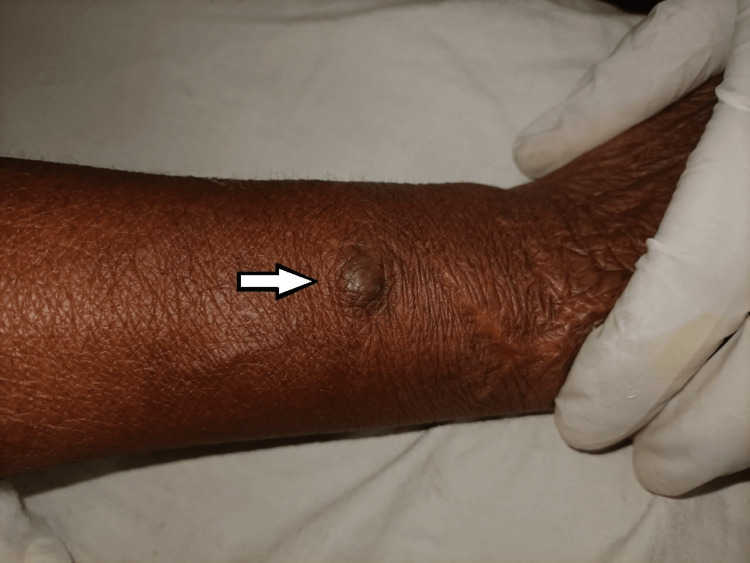
Clinical photograph showing the swelling over the dorsal aspect of left forearm (arrow).

Upon clinical examination, the swelling was firm, non-tender, and mobile. It measured approximately 20 x 14 mm and did not show any discharge or local rise in temperature (Figure 1).

The patient was further referred to the department of radiodiagnosis for an X-ray and ultrasound examination.

**Figure 2:**
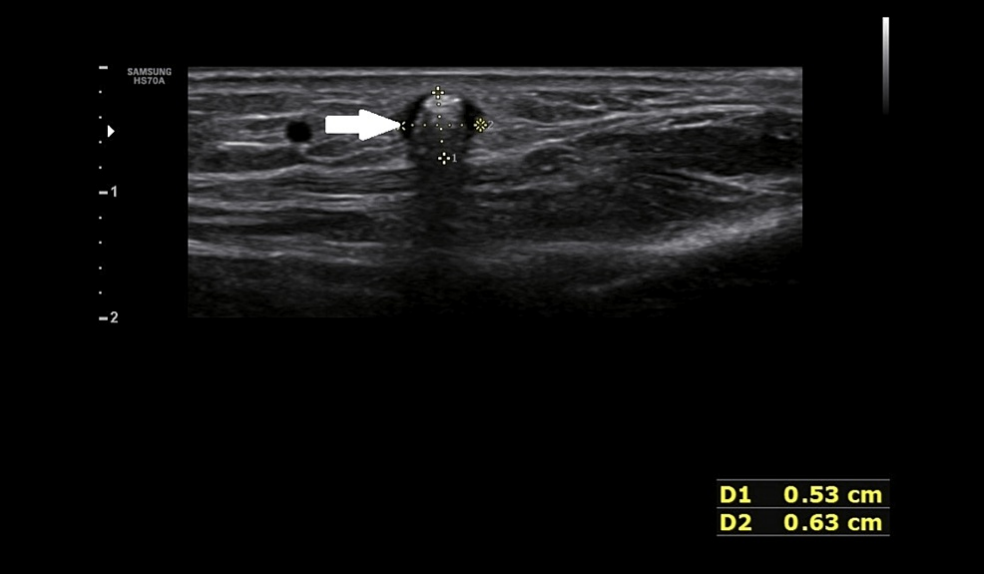
B mode ultrasonography image showing posterior acoustic shadowing (arrow)

On B mode ultrasound, two round-shaped hyperechoic foci was seen, with the larger one measuring 06 x 05 mm, on a transverse scan in the subcutaneous plane in the region of swelling (Figure 2).

**Figure 3:**
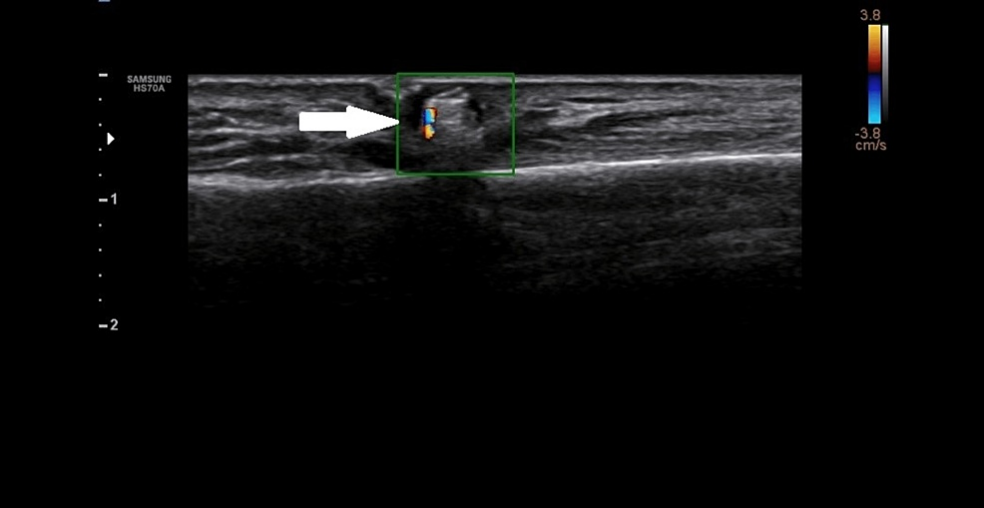
Colour Doppler image showing twinkling artifact (arrow)

These foci had posterior acoustic shadowing and showed twinkling artefacts on a colour flow ultrasound (Figure 3).

These echogenic foci were surrounded by hypoechoic hallow circumferentially and throughout their length.

**Figure 4:**
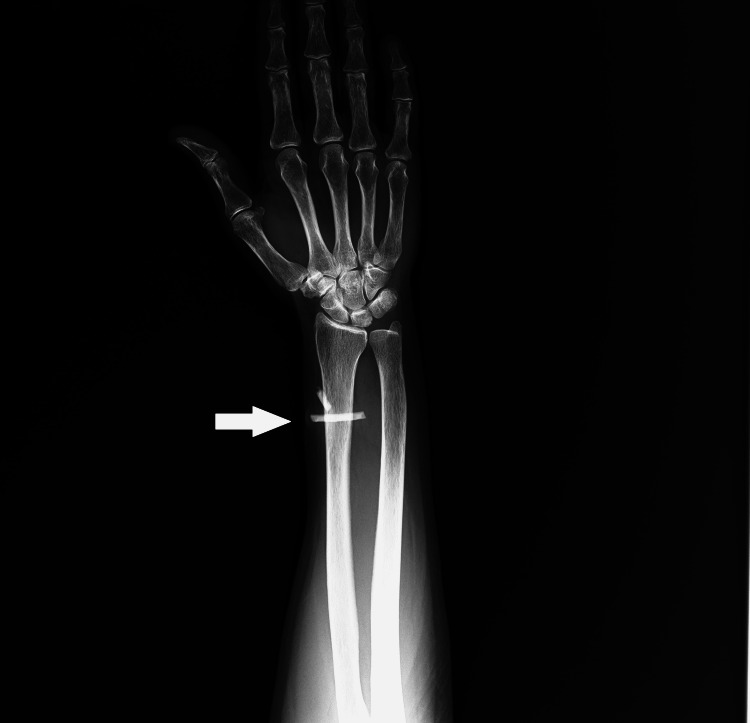
X ray image showing bangle pieces in distal forearm (arrow)

On X-ray, two radio-opacities were noted in the left distal forearm over the distal radius. This was consistent with broken fragments of a bangle in the subcutaneous plane (Figure 4).

On correlating the findings of both ultrasound and radiograph, the diagnosis of retained pieces of foreign body in the soft tissue of the left forearm was confirmed.

**Figure 5:**
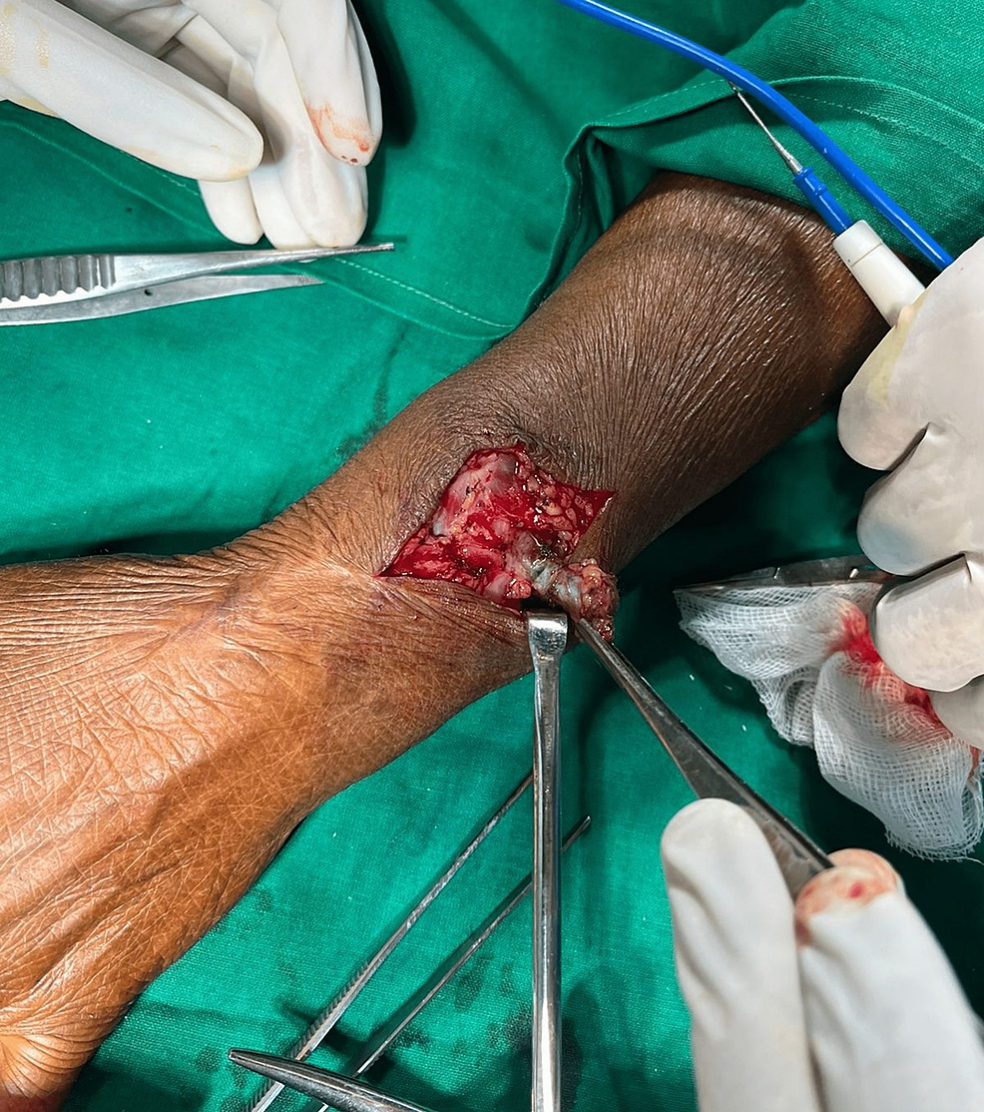
Intraoperative image while removing the foreign body

**Figure 6:**
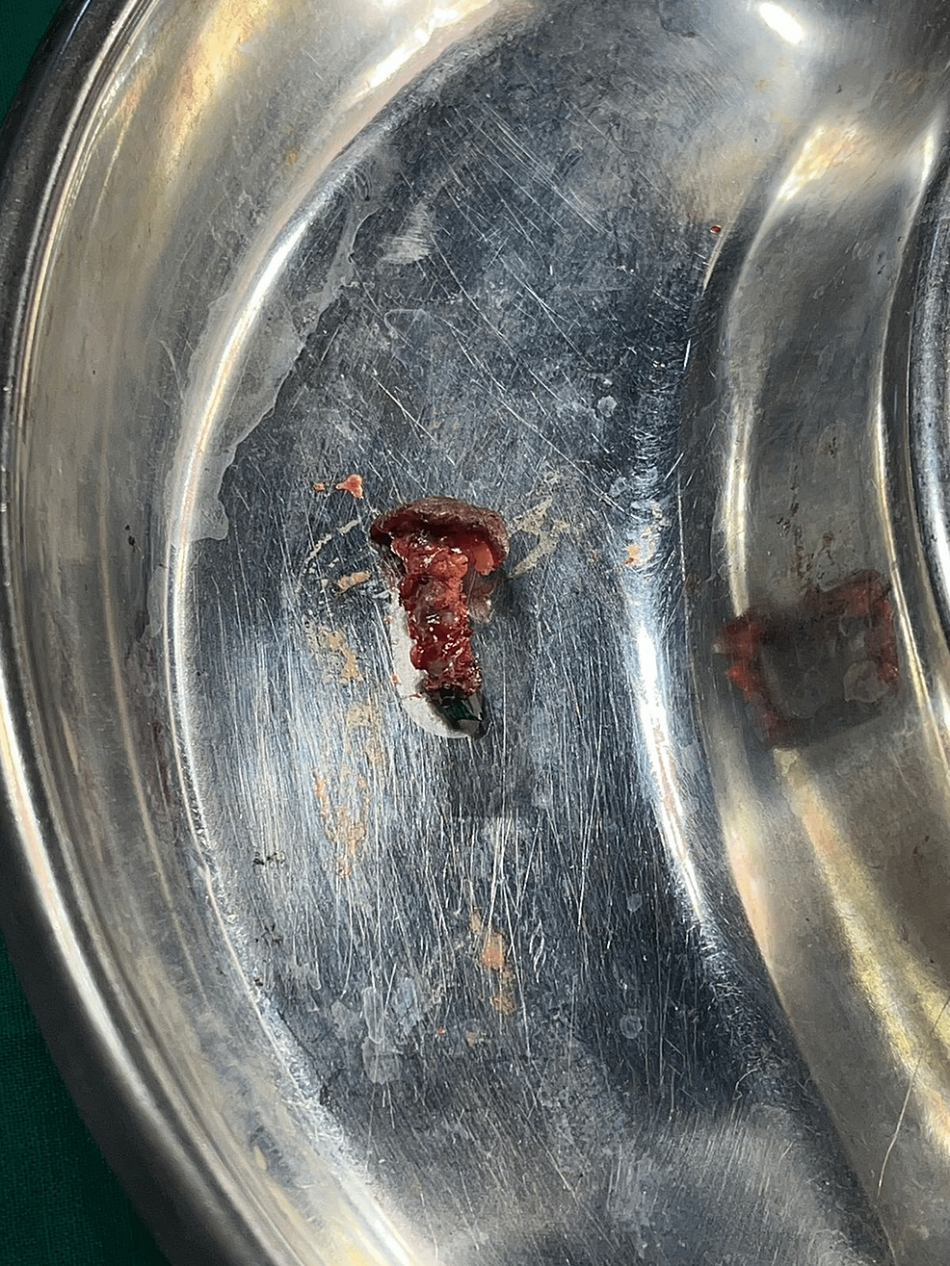
Retrieved bangle piece

She was operated on for the removal of the foreign bodies and the bangle fragments were removed from the swelling along with the adjacent soft tissue (Figures 5-6).

## Discussion

Foreign bodies are frequently impacted in the upper limbs and may remain there for a long time. These are identified during a routine examination through radiographic findings, but the absence of pain as a symptom can make the diagnosis of such lesions difficult [3]. When there is a clinical history of suspicion of a foreign body, plain radiographs should be immediately done. Foreign bodies which are radiopaque can be easily diagnosed but those which are radiolucent are always a challenge [4]. For ultrasound examination of suspected foreign bodies in the musculoskeletal system, the optimal choice is a high-frequency 7.5 Mhz or higher frequency linear array transducer. The foreign body’s composition decides its echogenicity, although foreign bodies in the musculoskeletal system generally exhibit increased echogenicity. Artefacts occurring deep in the foreign body are dependent on the surface characteristics of the object. Irregular or curved surfaces cause posterior shadowing with a "clean" appearance, while flat and smooth surfaces produce "dirty" shadowing, known as posterior reverberation or comet tail artefact [5]. Ultrasound also detects complications which arise from foreign bodies, such as infections, vascular issues, and tendon injuries [6]. Having knowledge of the exact location of a foreign body relative to skin surfaces, adjacent muscles, tendons, neurovascular bundles, and other vascular structures, which is obtained via ultrasound, allows more controlled surgical dissection. When the foreign body is near a vessel, Doppler sonography is a very useful tool for the detection of the position of the foreign body in relation to the vessel. When foreign bodies are located in hands and feet, a meticulous searching technique is required to detect small objects because they can be easily overlooked due to surrounding echogenic structures. More so when the foreign bodies are not lying parallel to the skin, the size of the foreign body makes it conspicuous rather than its composition. Linear transducers are helpful in its initial detection as a larger scanning area of a linear probe will accentuate its detection. High-frequency probes are useful in the detection as well as the measurement of the size of the foreign body as prominent echoes are visualized. Small-sector transducers are helpful in detection as well as intraoperative removal procedures [7]. CT scans also are useful for identifying foreign bodies. The appearance of foreign bodies on CT scans can vary. In acute cases it appears as areas of low density and in chronic cases where the foreign body has been retained for a prolonged time period it appears as high density areas. Dry foreign bodies initially contain air upon entry into the body, but they gradually absorb the surrounding exudate, which leads to an increase in density. Reactive lesions in the surrounding tissue appear hypodense-isodense compared to the muscles, and MRI is superior to CT for the detection of these lesions and determining their extent, especially when investigating soft tissues anywhere in the body. On MRI, foreign bodies appear as areas of low signal intensity or signal void in relation to the muscles, both on T1- and T2-weighted images [4]. Metallic artefacts are seen on MRIs even if a small quantity of metals like iron, cobalt or nickel are present. These metals become highly magnetized as soon as they are placed in a scanner. This causes distortion of the magnetic field around them causing artefacts. These metallic artefacts cause signal loss and distortion of images. MRI is contraindicated for suspected ferrous-based materials, nickel alloys, and most stainless steel materials foreign bodies [8].

## Conclusions

Clinicians should always consider the potential presence of a foreign object when evaluating recurring, unexplained infections or swelling. High frequency ultrasound has high sensitivity and specificity in diagnosing soft tissue foreign bodies. Sonographic guidance in case of foreign body can help in reduction of size of incision and deciding precise depth during removal of foreign body. Preoperative sonography greatly enhances detection and localization of foreign bodies as well as helps the surgeon in proper patient management.
